# Development of lactate‐related gene signature and prediction of overall survival and chemosensitivity in patients with colorectal cancer

**DOI:** 10.1002/cam4.5682

**Published:** 2023-02-12

**Authors:** Zhi Tong, Xinyu Wang, Sanbao Shi, Tiewei Hou, Guangrong Gao, Da Li, Yongqi Shan, Cheng Zhang

**Affiliations:** ^1^ Department of General Surgery General Hospital of Northern Theater Command (Formerly Called General Hospital of Shenyang Military Area) Shenyang China; ^2^ Postgraduate College China Medical University Shenyang China

**Keywords:** colorectal cancer, immune, lactate, nomogram, prognostic signature, tumor microenvironment

## Abstract

**Background:**

Colorectal cancer (CRC) is a malignant tumor of the digestive system that contains high levels of immune cells. Lactic acid, a major metabolite, plays a crucial role in tumor development, maintenance, and therapeutic response. However, the prognostic potential and therapeutic biomarker potential of lactate‐related genes (LRGs) in CRC patients remain to be elucidated.

**Methods:**

We collected the mRNA expression profile and clinical data of CRC patients from the Cancer Genome Atlas (TCGA) database and the GSE59382 cohort. Univariate Cox regression, Lasso regression and multivariate Cox regression analysis were used to construct the prognosis model. Combined with the risk score and important clinicopathological features, the nomogram was established. In addition, the relationship between risk score and immune infiltration, immune checkpoint gene expression, and drug sensitivity was investigated.

**Results:**

We constructed lactate‐related gene signatures (LRGS) based on four LRGs, which independently predicted the prognosis of CRC. Patients with different risk scores are found to have distinct immune status, tumor mutation load, immune response, and drug sensitivity. In addition, nomogram results determined by risk scores and clinical factors have higher predictive performance.

**Conclusion:**

We found that LRGS is a reliable biomarker for predicting clinical outcomes, evaluating immune infiltration and efficacy, and predicting the sensitivity to drugs in patients with CRC.

## Graphical abstract

Lactate‐related gene signature (LRGS) based on 4 lactate‐related genes were developed and validated in CRC patients to predict clinical outcomes and provide individualized treatment recommendations. Patients with higher LRGS showed worse outcomes in different cohorts. Higher LRGS indicate better immunotherapy response, while lower LRGS indicate better sensitivity to targeted drugs.

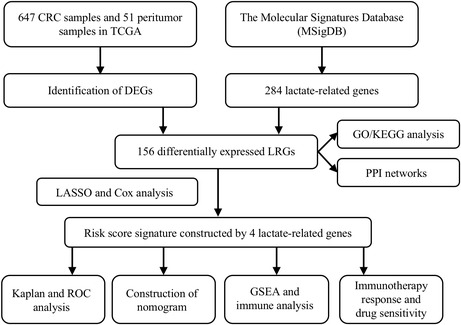

## 1 INTRODUCTION

Approximately 2 million colorectal cancer (CRC) cases and 940,000 related deaths occur each year; therefore, CRC is regarded to be the third most normal tumor as well as the second‐leading reason for cancer‐related deaths across the world.^1^ Nowadays, the treatment of CRC mainly consists surgical resection, chemotherapy, radiotherapy, immunotherapy, targeted therapy, and other methods.^2^ Even though these treatments are beneficial in patients with CRC, patients with advanced stage or those with metastasis often have a poor prognosis.^3^ Improving the prognosis of patients with CRC still becomes a challenge for clinicians. As a result, new biomarkers for CRC should be developed to predict clinical outcomes and provide individualized treatment.

A recent study has linked metabolism to tumor development,^4^ and metabolic reprogramming has become a hallmark of cancer. Because of the nutrient‐poor environment of tumor cells (e.g., an environment lacking in oxygen), they often use metabolic reprogramming to meet their energy needs. Tumor cells adapt to the complex tumor microenvironment (TME) by reprogramming glucose,^5^ fatty acid,^6^ and amino acid metabolism^7^ in order to promote their proliferation and growth. The levels of lactate, a common metabolite, are significantly elevated in malignant tumors.^8^ Even under oxygen‐sufficient conditions, cancer cells can accomplish lactate accumulation through the glycolytic and glutaminolysis pathways.^9^, ^10^ Cells excrete lactate through monocarboxylate transporters to maintain a low lactate intracellular environment and promote continued lactate production. The acidic TME favors tumor growth and development.^11^

Lactate promotes tumor growth and development by providing energy to tumor cells, promoting tumor angiogenesis, and participating in immune escape and chemoresistance. In gliomas, lactate promotes tumor angiogenesis through the activation of hypoxia‐inducible factor in normoxic endothelial cells.^12^ In breast cancer, lactate induces programmed cell death ligand‐1 (PD‐L1) through the activation of GPR81 (a G‐protein‐coupled receptor), thereby leading to immune escape.^13^ The lactate/BDNF/TrkB signaling pathway promotes drug resistance in gastric cancer by mediating epithelial‐stromal cell interactions.^14^ In TME, the accumulation of lactate affects the phenotype and function of immune cells, leading to the development of immune system tolerance and immunosuppression.^15^ In breast cancer, tumor‐produced lactate triggers the polarization of M2 macrophages through the activation of the ERK/STAT3 signaling pathway.^16^ A study on CRC revealed that lactic acid induces NK cell apoptosis by lowering the pH of TME.^17^ In addition, lactic acid suppresses immune function by damaging cytotoxic T lymphocytes.^18^

Considering the important role of lactate in tumorigenesis and immunosuppressive tumor microenvironment, cancer therapy targeting its metabolism should be developed. The construction of genetic signatures to determine the prognosis of cancer patients is regarded to be a feasible strategy; nevertheless, no predictive model has been constructed to investigate the mechanism of action of lactic acid‐related genes in CRC. The Cancer Genome Atlas (TCGA) and the Gene Expression Omnibus (GEO) are two of the most widely used public databases, and their mining yields a wealth of cancer information.^19^, ^20^ This work extracted data from the TCGA database and analyzed the expression profile of all patients with CRC with the aim of constructing a lactate‐related gene signature (LRGS) for the prediction of survival outcomes. These results were validated by the GEO database. Furthermore, we analyzed the immunotherapy response and chemotherapeutic drug sensitivity in different subgroups of patients. The results indicated that LRGS can predict highly accurate prognostic outcomes in patients with CRC and guide such patients to choose the best immunotherapy and chemotherapy regimens.

## 2 MATERIALS AND METHODS

### 2.1 Data collection

We downloaded mRNA expression data, clinical data, and mutation profiles of tumor and normal tissues of all patients suffering from colon and rectal cancer from the TCGA database (https://portal.gdc.cancer.gov). Besides, the downloaded data were composed of mRNA expression data of 480 colon cancer tissues, 41 normal colon tissues, 167 rectal cancer tissues, and 10 normal rectal tissues; clinical data of 630 patients with CRC; and mutation data of 582 patients with CRC. We randomly divided the patients with CRC into the training group (*n* = 350) and the test group (*n* = 223) at the ratio of 6:4. In addition, we downloaded the GSE39582 (*n* = 585) dataset from the Gene Expression Omnibus (GEO) database (https://www.ncbi.nlm.nih.gov/). In order to determine the stability of the prognostic model, the dataset was applied to be an external validation set.^21^ The clinical and pathological characteristics of colorectal cancer patients are summarized in Table 1.

**TABLE 1:** Clinical and pathological features of patients with CRC.

Variables	TCGA cohort (*N* = 582)	GSE39582 (*N* = 585)
Age (years)		
≤65	260	228
>65	322	356
NA	0	1
Gender		
Male	317	322
Female	265	263
NA	0	
Stage		
I–II	309	313
III–IV	253	270
NA	20	2
T		
1–2	122	65
3–4	458	498
NA	2	22
N		
0	328	314
1–3	251	243
NA	3	28
M		
0	433	499
1	84	61
NA	65	25

### 2.2 Lactate metabolism‐related genes (LRGs)

LRGs were downloaded from the Molecular Signatures database (MSigDB; https://www.gsea‐msigdb.org/gsea/msigdb/index.jsp).^22^ A total of 458 genes involved in nine signaling pathways, including lactate transmembrane transport, lactate dehydrogenase activity, lactate transmembrane transporter activity, abnormal brain lactate levels (detected by magnetic resonance spectroscopy), abnormal lactate dehydrogenase level, elevated lactate‐to‐pyruvate ratio, increased circulating lactate dehydrogenase concentration, increased cerebrospinal fluid lactate, and increased serum lactate were retrieved. We deleted duplicate genes to obtain a total of 284 genes.

### 2.3 Differential expression of LRGs and functional enrichment analysis

This study applied the R package “edgeR”^23^ to determine the differential level of LRGs in tumor and normal tissues, with the threshold for |log2 fold change (FC)| > 1.0 and *p* < 0.05. Additionally, the Kyoto encyclopedia of genes and genomes (KEGG) and Gene ontology (GO) analyses were performed for annotating differentially expressed proteins. The GO and KEGG enrichment analyses of LRGs were conducted with the “ClusterProfiler” of the R package.^24^ We adopted STRING database (https://string‐db.org/) for creating the PPIs for LRGs, and the results were visualized with Cytoscape.^25^, ^26^

### 2.4 Construction and validation of LRGS

First, we used the univariate Cox regression analysis for screening LRGs with a significant prognostic value. To avoid overfitting, the “glmnet” package was applied to further carry out LASSO Cox regression (1000 iterations) on the selected genes.^27^ Finally, the selected genes were explored by adopting the multivariate Cox regression analysis in order to establish an LRG‐based prognostic model. On the basis of the formula: risk score = ∑(coefficient × expression of signature gene_i_), the risk score was computed. In addition, patients were categorized into the high‐risk (>median) and low‐risk (<median) groups in line with the median risk score. Principal component analysis (PCA) and T‐distribution random neighborhood embedding (T‐SNE) were employed with the aim of evaluating the distribution of the model in different subgroups. By adopting the R package “scatterplot3D”, PCA was conducted.^28^ In addition, T‐SNE was conducted by adopting the R package “Rtsne”.^29^

Kaplan–Meier analysis, time‐dependent receiver operating characteristic (ROC) analysis, risk score distributions, and heat map construction were carried out for different subgroups of patients using data from training set, internal validation set, and external validation set to test the performance of the model in predicting clinical outcomes.

We adopted univariate and multivariate cox regression analyses for determining the independence of the model for prognostic prediction. In addition, survival analysis was conducted for subgroups of patients with different clinical characteristics to assess the model's applicability.

### 2.5 Gene set enrichment analysis (GSEA) of the LRGS

With the purpose of elucidating the important functional phenotypes that differ between the high‐risk and low‐risk groups, the current work downloaded h.all.v7.5.1.symbols.gmt from the GSEA database (http://www.gsea‐msigdb.org/gsea/index.jsp). Normalized mRNA expression data were explored using GSEA (version 4.2.3). We set the following standards for the analysis: genetic size ≥15, |standardized enrichment fraction (NES)| > 1.5, nominal *p*‐values (NOM) *p* < 0.05, and FDR *q*‐value <0.25.

### 2.6 Nomogram development for predicting survival

With the purpose of determining the 1‐year, 3‐year, and 5‐year survival rates of patients undergoing CRC, we employed R packages “rms” and “regplot” to construct a nomogram using LRGS and important clinicopathological parameters (age, sex, and tumor stage).^30^ In addition, calibration curves and ROC curves were constructed with the aim of assessing the availability of the nomogram.

### 2.7 Mutation burden and evaluation of the therapeutic effect

We attempted to calculate the tumor mutation burden (TMB) for each colon adenocarcinoma and rectum adenocarcinoma sample in the TCGA to evaluate differences in somatic mutation data between the high‐risk and low‐risk groups; the findings were visualized according to the R package “maftools”.^31^ Tests using different immune checkpoint expression levels were used together with models to calculate the treatment sensitivity in both risk groups. To analyze chemotherapeutic drug sensitivity in different risk groups, the present study applied the “pRRophetic” software package with the aim of exploring the level of various drug target genes in different subgroups by using the ridge regression evaluation of semi‐inhibitory concentration (IC50).

### 2.8 Immunoinfiltration analysis

We used CIBERSORT,^32^ quanTIseq,^33^ McP‐counter,^34^ Xcell,^35^ ESTIMATE,^36^ IPS,^37^ and EPIC^38^ algorithms to present the connection between risk score and tumor‐infiltrating immune cells. Moreover, we evaluated differences in stroma score, immune score, ESTIMATE score, and tumor purity between the two groups with the ESTIMATE algorithm in line with the gene expression profiles of the two groups.

### 2.9 Cancer immune response evaluation

The anticancer immune response of the body or the progressive events of the cancer‐immune cycle consists of the release of cancer cell antigens, cancer antigen presentation, the initiation and activation of immune cells, immune cell transportation to the tumor, immune cell invasion of the tumor, and the recognition and killing of cancer cells by T cells. We generated activity scores for each immunization step based on CRC mRNA expression using the Tracer Immunophenotype (TIP; http://biocc.hrbmu.edu.cn/TIP/index.jsp).^39^ Subsequently, this study attempted to compare the differences in the scores across the seven steps with the purpose of exploring the anti‐cancer immune status as well as the ratio of tumor‐infiltrating immune cells between the different groups.

### 2.10 Tissue samples

Colon and rectal tumor tissues and pan‐cancer tissues of 30 patients undergoing surgical treatment for CRC were obtained from the Department of General Surgery of Northern Theater Command General Hospital. Before surgery, none of the patients could receive chemotherapy or other treatment. Then, all the involved samples were kept in a −80°C refrigerator until real‐time PCR (qRT‐PCR) was analyzed. All the involved patients signed a written informed consent form. The approval of the current work was acquired by the Ethics Committee of Northern Theater General Hospital.

### 2.11 Quantitative real‐time PCR

Total mRNA from colon and rectal tumor tissues was extracted with the use of TRIzol (NucleoZOL, #740404.200). Reverse transcription was performed using a kit (NovoScript, #E047‐01B). qPCR was carried out based on SYBR qPCR master mix (NovoStart, #E096‐01B) in a total volume of 20 μL by adopting the TL988 Real‐Time PCR system. qRT‐PCR primer sequences are presented in Table S1. We calculated relative quantification values of RNA with the application of the 2^−ΔΔCt^ method.

### 2.12 Statistical analysis

All data were explored and visualized with the use of the R 4.1.2 software. We used the two‐tailed *t*‐test and one‐way analysis of variance (ANOVA) with the aim of determining differences between and among groups. This study applied the chi‐squared test to investigate correlations between the classified data. We adopted Kaplan–Meier analysis for calculating the overall survival (OS). It was shown that a *p*‐value of <0.05 was regarded to present statistical significance.

## 3 RESULTS

### 3.1 LRG identification and functional enrichment analysis

**FIGURE 1:**
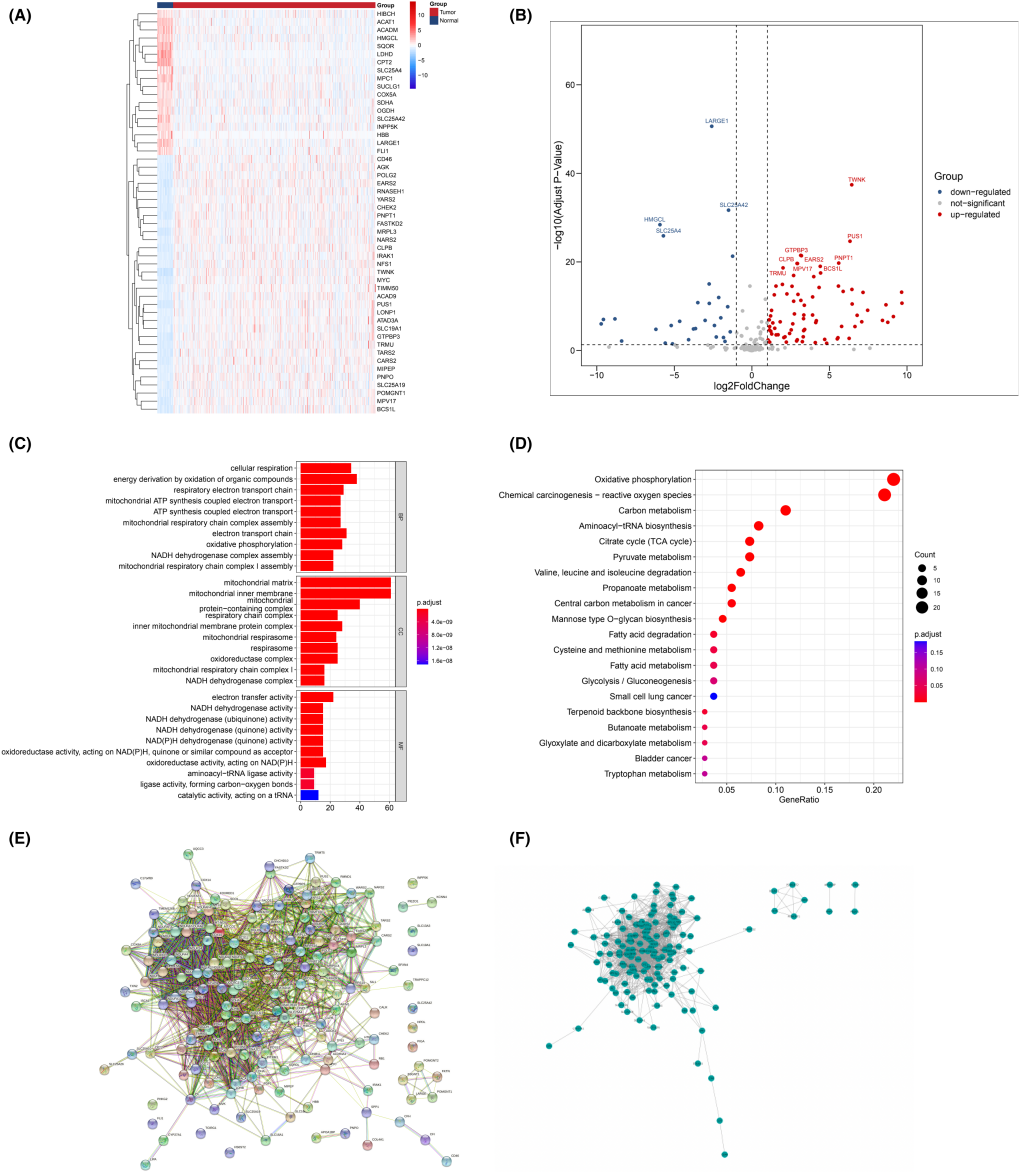
CRC identification and enrichment of the differences in gene analysis. (A) Volcanograms indicating downregulated and upregulated LRGs. (B) Heat map indicating 50 LRGs, with red representing upregulated genes and blue downregulated genes. (C) Histogram of Gene Ontology enrichment. (D) Bubble diagram for KEGG enrichment analysis. (E) Protein–protein interaction (PPI) networks with differential expression of LRGs. (F) Differential expression of LRGs “MCODE” module.

We used differential gene screening to identify 156 differentially expressed LRGs consisting of 103 upregulated genes and 53 downregulated genes. Figure 1A depicts a heat map revealing differential gene expression in CRC and normal samples. Figure 1B illustrates a volcano diagram revealing differential gene expression based on downregulated and upregulated genes.

We analyzed the data by using functional enrichment analysis. The biological processes (BP) enriched by differentially expressed genes included cellular respiration, energy generation by the oxidation of organic compounds, respiratory electron transport chain, ATP synthesis coupled electron transport, and mitochondrial respiratory chain complex assembly. The cellular components (CC) enriched by differentially expressed genes included mitochondrial matrix, mitochondrial intima, mitochondria, protein complex, and respiratory chain complex. The molecular functions (MF) enriched by differentially expressed genes included electron transfer activity, NADH dehydrogenase activity, NADH dehydrogenase, and NADPH dehydrogenase activity (Figure 1C). The KEGG analysis revealed the differentially expressed gene‐enriched pathways to be those involved in oxidative phosphorylation, chemical carcinogenesis‐reactive oxygen species, citrate cycle, carbon metabolism, pyruvate metabolism, aminoacyl‐tRNA biosynthesis, valine degradation, leucine degradation, and isoleucine degradation, propanoate metabolism, mannose type O‐glycan biosynthesis, central carbon metabolism in cancer, fatty acid degradation, terpenoid backbone biosynthesis, cysteine and methionine metabolism, butanoate metabolism, fatty acid metabolism, glyoxylate and dicarboxylate metabolism, glycolysis/gluconeogenesis, bladder cancer, tryptophan metabolism, and small cell lung cancer (Figure 1D). PPI networks were constructed for the above‐mentioned differentially expressed LRGs, and the MCODE algorithm was used to identify the most important modules (Figure 1E,F). According to the results, the LRGs were mainly related to metabolic processes and oxidation reaction, suggesting that the cells produce lactic acid if metabolic and oxidative reactions are high.

### 3.2 LRGS development

**FIGURE 2:**
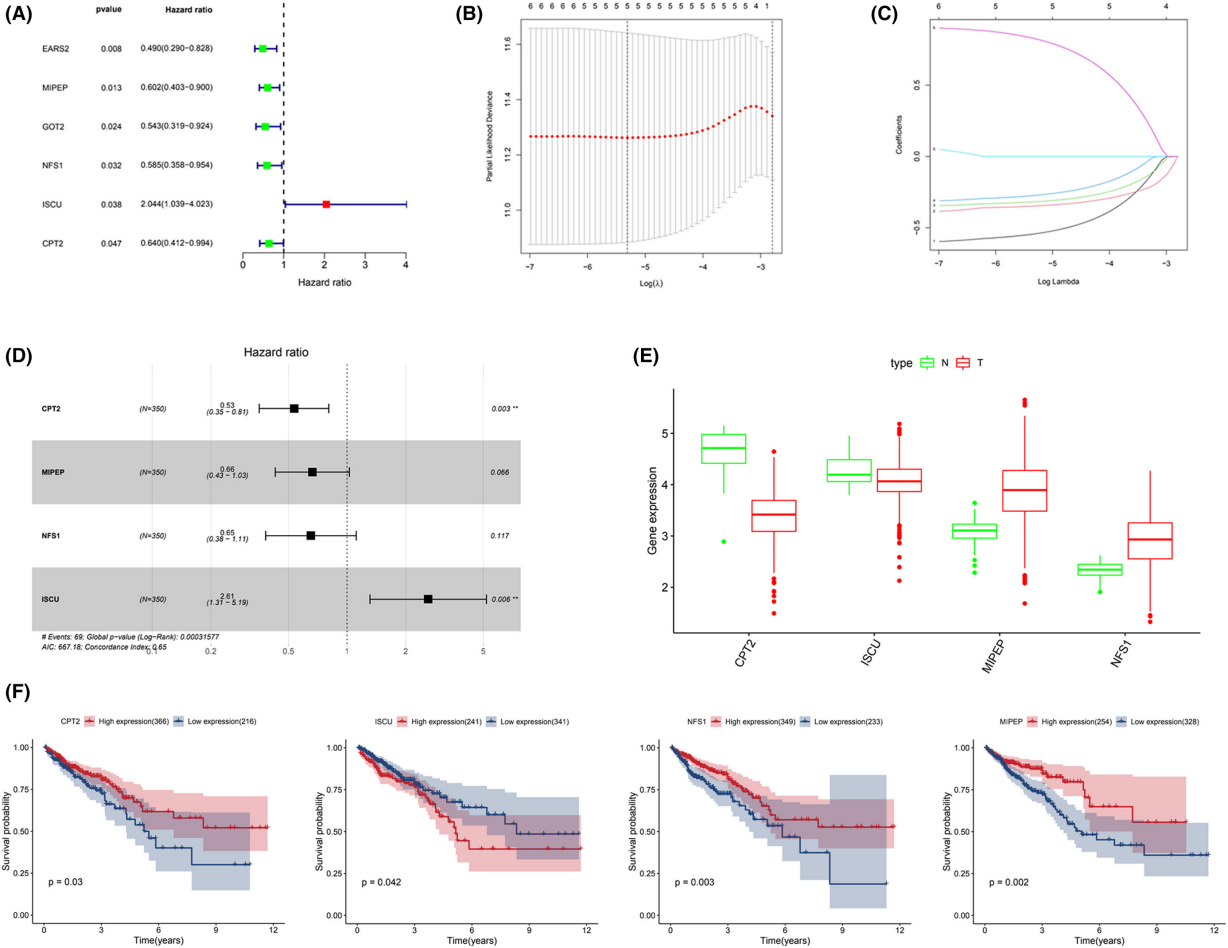
Construction and composition analysis of LRGS. (A) Six LRGs were chosen by using univariate Cox regression analysis to influence the prognosis. (B) Identification of optimal parameters in the Lasso model. (C) Distribution of Lasso coefficients. (D) Forest maps of the four genes that constitute LRGS. (E) Expressions of four target genes were analyzed using the TCGA database. (F) Kaplan–Meier analysis of four target genes by using the TCGA database.

The univariate Cox regression analysis of LRGs demonstrated that six genes were obviously correlated with OS (Figure 2A). With the aim of removing the effect of overfitting, five genes were preserved using the LASSO analysis. Subsequently, four genes (carnitine palmityl transferase 2 [*CPT2*], mitochondrial intermediate peptidase [*MIPEP*], cysteine desulfurase [*NFS1*], and iron–sulfur cluster assembly enzyme [*ISCU*]) were used to construct the LRGS by adopting the multivariate Cox regression analysis (Figure 2B,C). In addition, among the four genes, three were protective genes (*CPT2*, *MIPEP*, and *NFS1*), and one was a risk gene (*ISCU*; Figure 2D). According to the analysis of the TCGA data, the expression of *MIPEP* and *NFS1* was higher in tumor tissues in comparison with that in normal tissues; however, the expression of *CPT2* and *ISCU* was lower in tumor tissues in relative to that in normal tissues (Figure 2E). The qRT‐PCR results of *CPT2*, *ISCU*, and *MIPEP* validated the aforementioned results; however, the qRT‐PCR results did not reveal an obvious difference in the expression of *NFS1* between the tumor and normal tissues (Figure S1). Obviously, the Kaplan–Meier survival curves presented poor prognosis in patients undergoing a low expression of *CPT2*, *MIPEP*, and *NFS1* and a high expression of *ISCU* (Figure 2F).

### 3.3 Prognostic significance of LRGS

**FIGURE 3:**
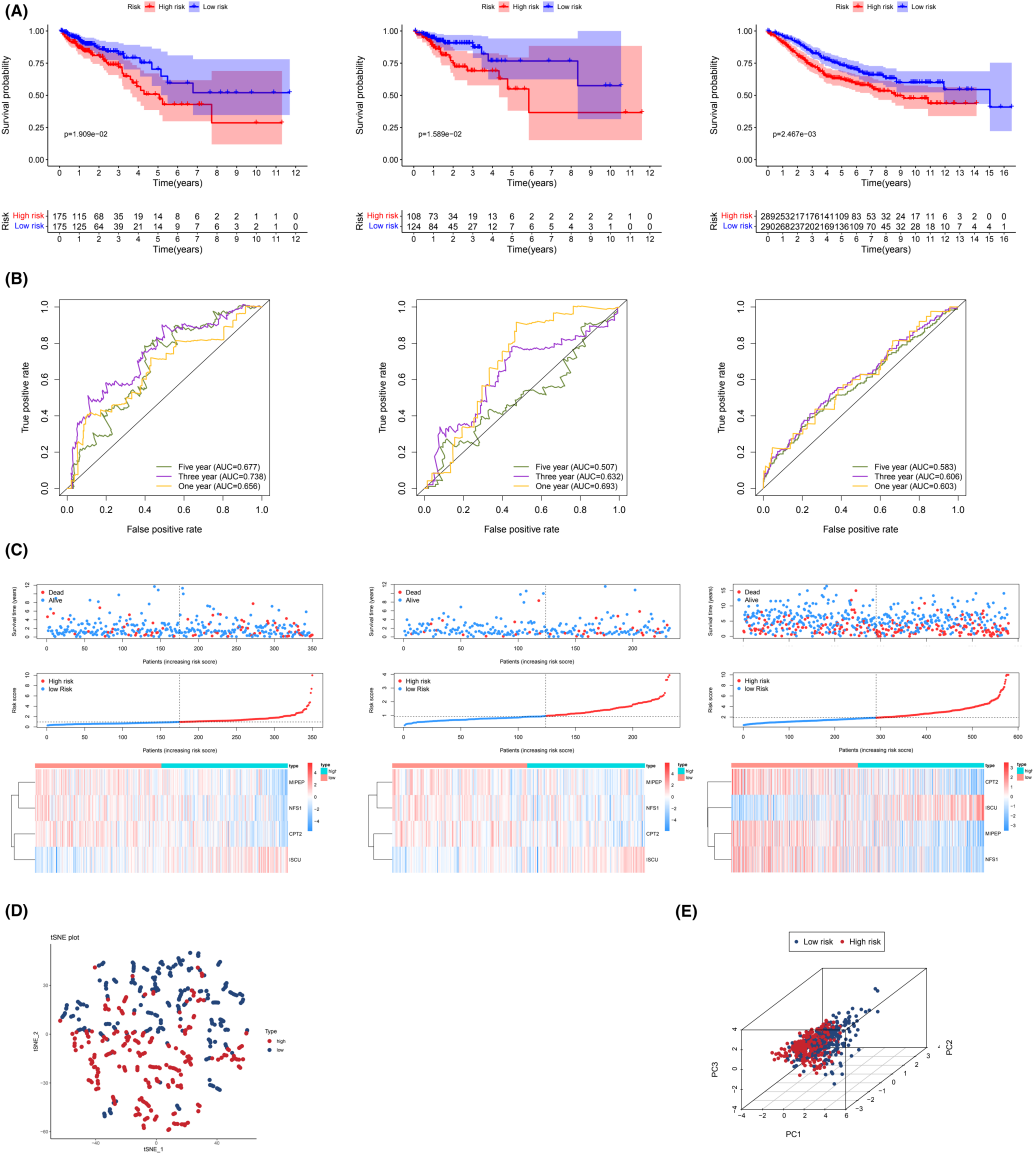
Validation of LRGS. (A) Kaplan–Meier analysis of LRGS in three cohorts. (B) ROC curve analysis of LRGS at 1, 3, and 5 years in three cohorts. (C) The survival status analysis, score distribution analysis, and four target gene expression heat maps of LRGS in three cohorts (D) t‐distributed stochastic neighbor embedding (t‐SNE) analysis of TCGA queue. (E) PCA analysis of TCGA queue.

Considering the median risk score, this study classified patients into the low‐risk and high‐risk groups. Besides, in the training set, internal validation set, and external validation set, the survival of the low‐risk group was higher when compared with that of the high‐risk group. The p values of the Kaplan–Meier survival curves were 0.019, 0.016, and 0.002, respectively (Figure 3A). In the time‐dependent ROC analysis of the training set, the 1, 3, and 5‐year survival rates were 0.656, 0.738, and 0.677, respectively (Figure 3B); similar results were demonstrated by the validation set and external validation set (Figure 3A,B). We combined the gene expression versus survival data of all patients in the training and validation set to redraw the ROC curves with acceptable AUCs of 0.650, 0.655, and 0.627 for 1, 3, and 5 years, respectively (Figure S2). The risk score and survival status diagrams revealed that the risk score was negatively related to survival time (Figure 3C). The heat map revealed the differential expression of four key genes in two LRGS subgroups (Figure 3C). According to the PCA and t‐SNE analysis results, the patients in different risk groups were grouped into two directions (Figure 3D,E).

**FIGURE 4:**
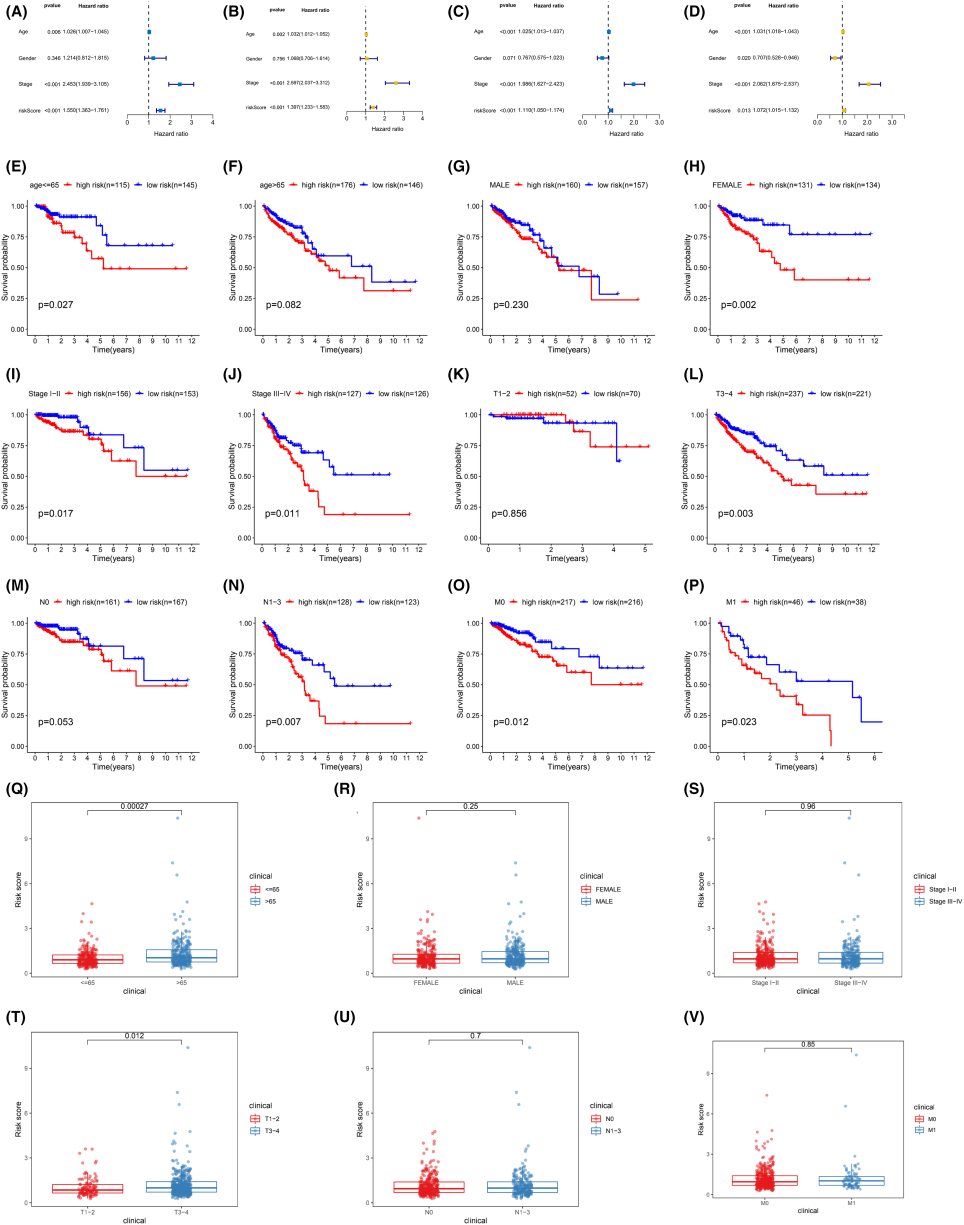
Univariate and multivariate Cox analyses of LRGS and other clinical characteristics in different cohorts. (A, B) TCGA. (C, D) GEO. Kaplan–Meier analysis of LRGS stratified by different clinical case parameters (E, F) Age. (G, H) Sex. (I, J) Stage. (K, L) T stage. (M, N) N stage. (O, P) M stage. Analyses based on the relationship between LRGS and different clinicopathological features (Q) Age. (R) Sex. (S) Tumor stage. (T) T stage. (U) N stage. (V) M stage.

According to the combined univariate and multivariate Cox regression analysis, the LRGS model predicted the OS independently (HR 1.550, 95% CI 1.363–1.761, P 2.44 E‐06) (Figure 4A,B); the result was confirmed by analyzing the GEO cohort (Figure 4C,D). Subsequently, we stratified patients considering their clinical characteristics to identify the patient groups appropriate for our model. The results indicated that the LRGS model predicted patients' prognosis in all subgroups except in subgroups with older patients aged above 65 years, men, and the cancer stages of T1, T2, and N0 (Figure 4E–P). In addition, we evaluated the association of LRGS with clinicopathological factors (age, sex, tumor stage, T stage, and N stage together with M stage) retrieved from the TCGA cohort. Based on the findings, the LRGS model correlated with age and T stage but not with sex, tumor stage, M stage, or N stage (Figure 4Q–V).

### 3.4 GSEA

**FIGURE 5:**
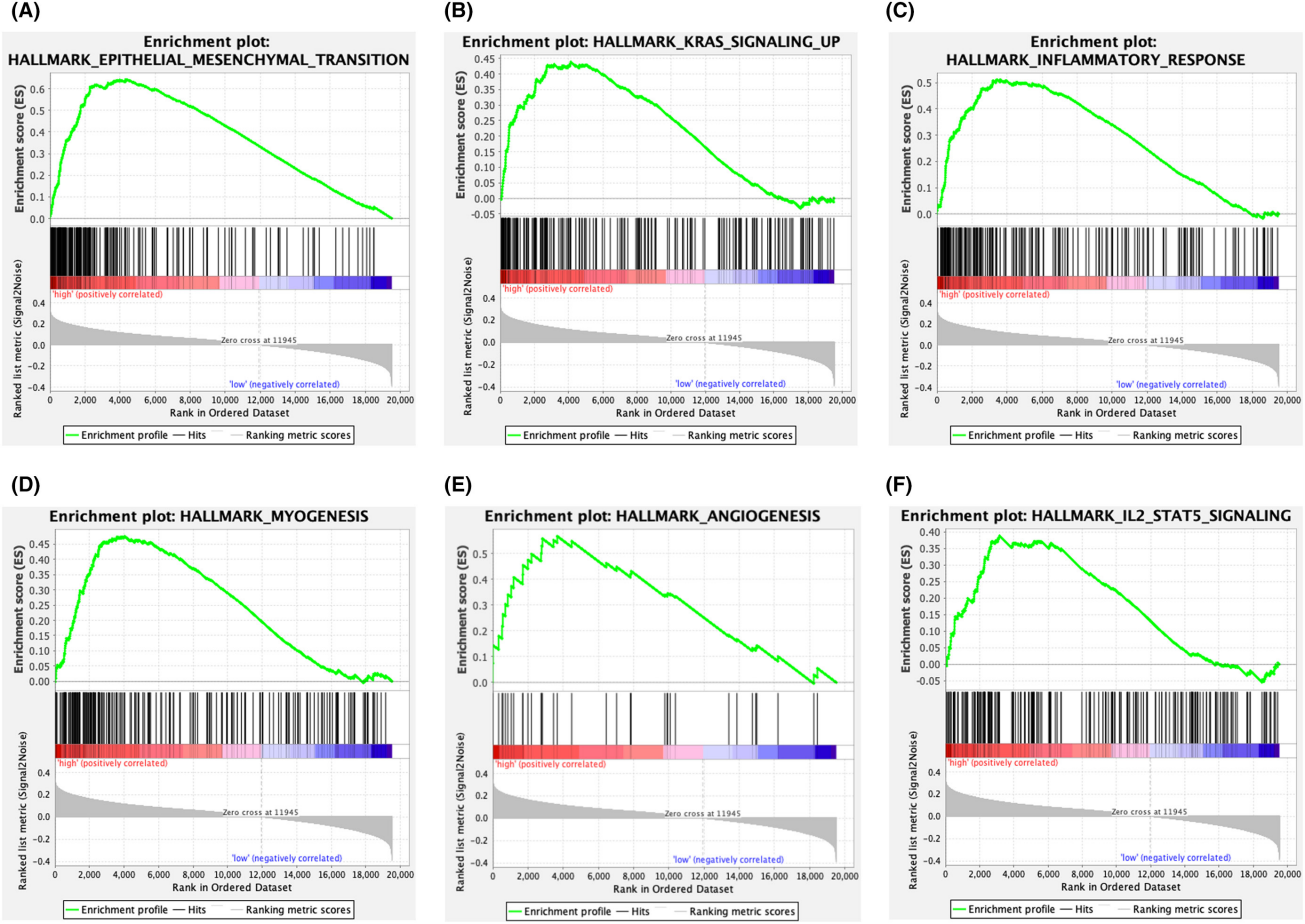
Relationship between LRGS and GSEA analysis. (A–F) Gene set enrichment analysis (GSEA).

In order to study the molecular mechanism of lactic acid in CRC, the GSEA software was used. The enrichment results of marker genes revealed that the extracellular matrix (ECM), KRAS signaling pathway, inflammatory response, myogenesis, angiogenesis, and IL‐2 STAT5 signaling were enhanced in the high‐risk group (Figure 5A–F).

### 3.5 Nomogram development

**FIGURE 6:**
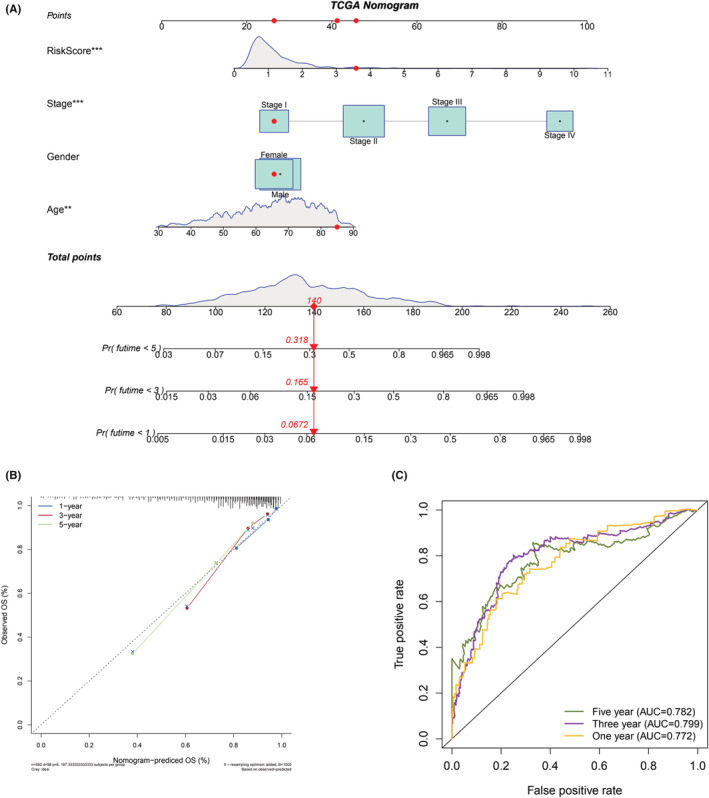
Construction and evaluation of nomogram. (A) A nomogram on the basis of LRGS and clinicopathological parameters. (B) Calibration diagram of the nomogram adopted for predicting overall survival at 1, 3. And 5 years. (C) ROC curve of the nomogram.

To make the accurate prediction of the OS, we combined age, sex, tumor stage, and LRGS for constructing a nomogram (Figure 6A). Each variable was assigned a score, and depending on the total score, the 1, 3, and 5‐year survivals were estimated for those with CRC. Obviously, the calibration curve indicated an optimal consistency between the predicted and actual values (Figure 6B). On the basis of the ROC curve of the nomogram, we determined the AUC of 1 year, 3 years, and 5 years to be 0.772, 0.799, and 0.782, respectively; these values indicated the efficiency of LRGS in OS prediction (Figure 6C). The obtained findings indicate that the nomogram features high accuracy and can be applied to clinical practice.

### 3.6 Association with TMB

**FIGURE 7:**
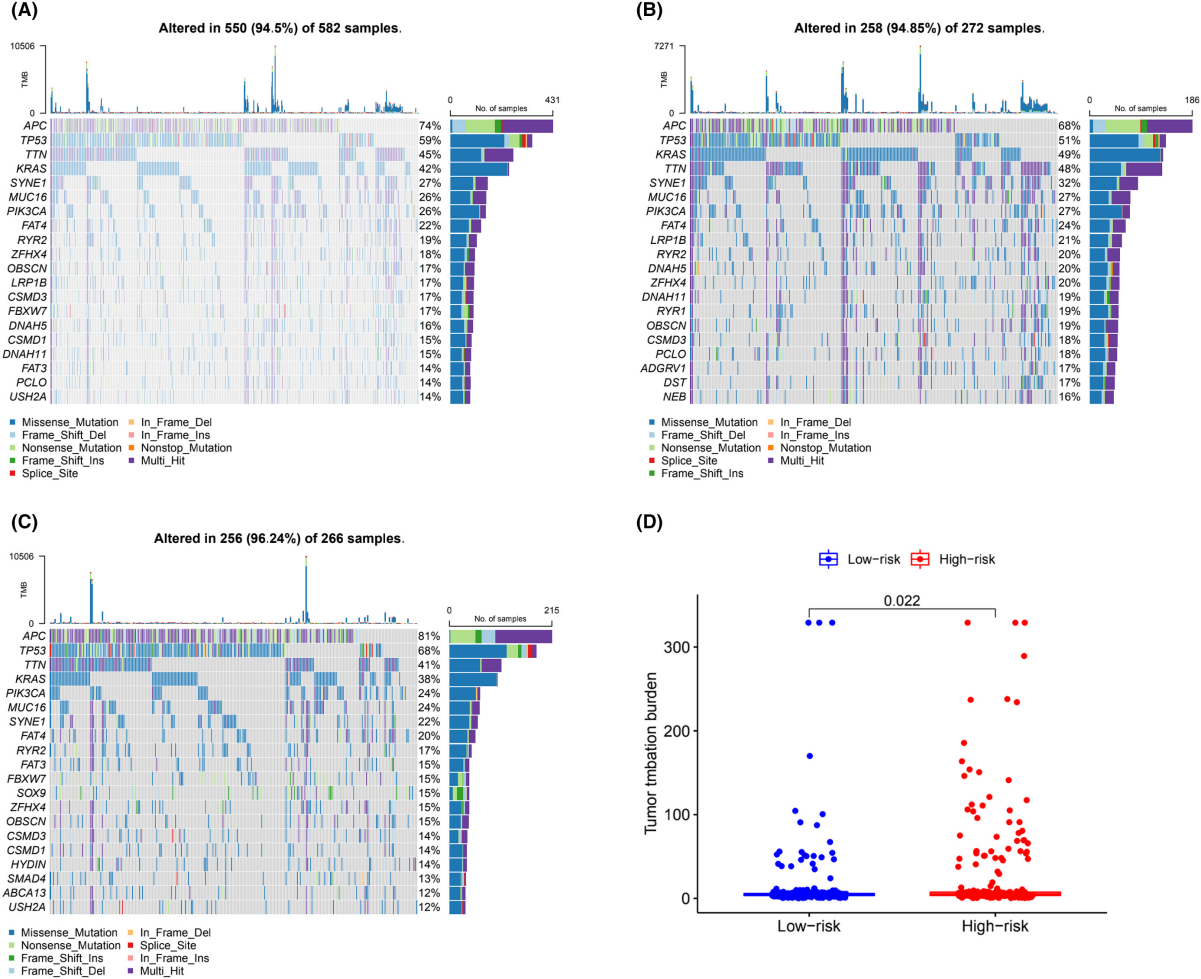
Relationship between LRGS and TMB. (A) Waterfall map of mutated genes in patients with CRC. (B) Waterfall map of mutant genes in LRGS‐high group. (C) Waterfall map of mutant genes in LRGS‐low group.(D) Difference in TMB between the two subgroups of LRGS.

We downloaded the mutation data of all patients undergoing CRC from the TCGA and calculated TMB for each patient. In the whole CRC dataset, the top 10 mutated genes were *APC*, *TP53*, *TTN*, *KRAS*, *SYNE1*, *MUC16*, *PIK3CA*, *FAT4*, *RYR2*, and *ZFHX4* (Figure 7A). Visualization results obtained from the analysis of somatic mutations in the two groups are provided in Figure 7B,C. With the exception of mutations in *APC* and *TP53*, mutations in critical genes were higher in the high‐risk group. A further investigation of TMB proved that TMB was higher in the high‐risk group (Figure 7D).

### 3.7 Analysis of TMEs

**FIGURE 8:**
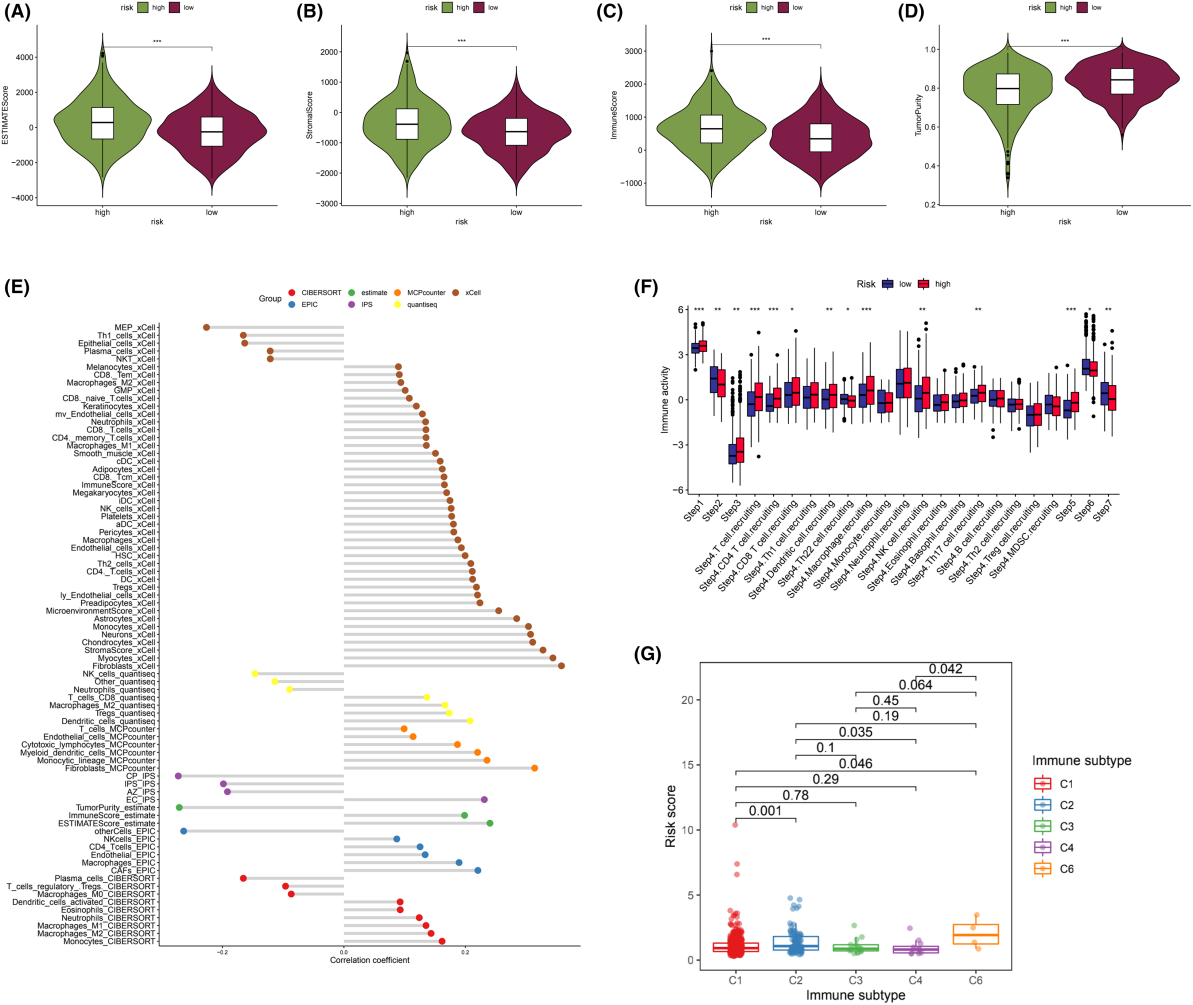
TME and immunoassay of LRGS. Differences in ESTIMATE score (A), tumor stroma score (B), immunity score (C), and tumor purity score (D) between the LRGS‐high and LRGS‐low groups, *p < 0.05; **p < 0.01; ***p < 0.001; ns, no sense. (E) Differences in immune‐related molecules and immune‐related cell types between the two subgroups of LRGS calculated using seven immune algorithms. (F) Existing difference in the abundance of immune cells between the LRGS‐high and LRGS‐low groups during antitumor process. (G) Relationship between LRGS and immune subtypes.

Various cells in the TME influence tumor progression, therapeutic effect, and treatment. Stroma score, immune score, ESTIMATE score, and tumor purity were computed for the two subgroups by using the ESTIMATE algorithm (Figure 8A–D). In relative to the low‐risk group, the high‐risk group generated a higher immune score, stroma score, and ESTIMATE score yet lower tumor purity (*p* < 0.05). In accordance with the obtained findings, we came to the conclusion that the risk score may show relationship to immune cell infiltration.

With the purpose of investigating the association between LRGS and CRC immune invasion, this study used CIBERSORT, quanTIseq, McP‐counter, xCELL, ESTIMATE, IPS, and EPIC algorithms to compare differences in immune cells and immune components between the two groups (Figure 8E). A total of 63 components showed positive relationship to the risk score, whereas the remaining 16 components were negatively correlated. We used the TIP analysis to compare the immune activity scores between the two groups at each step (Figure 8F). According to the obtained findings, the high‐risk group exhibited a higher level of immune cell infiltration in comparison with the low‐risk group.

Six pan‐cancer immune subtypes have been identified, with the prognosis of each immune subtype being different. LRGS and immune typing were correlated (Figure 8G); therefore, patients with C2 and C6 subtypes of CRC had higher risk scores. By contrast, most low‐risk patients with CRC had C1, C3, and C4 subtypes. The patients with the C3 subtype had a better prognosis, whereas the patients undergoing the C6 subtype had a worse prognosis. These findings complement our previous results and indicate that the characteristics of the CRC immune environment affect tumor development and progression differently.

### 3.8 LRGS and treatment

**FIGURE 9:**
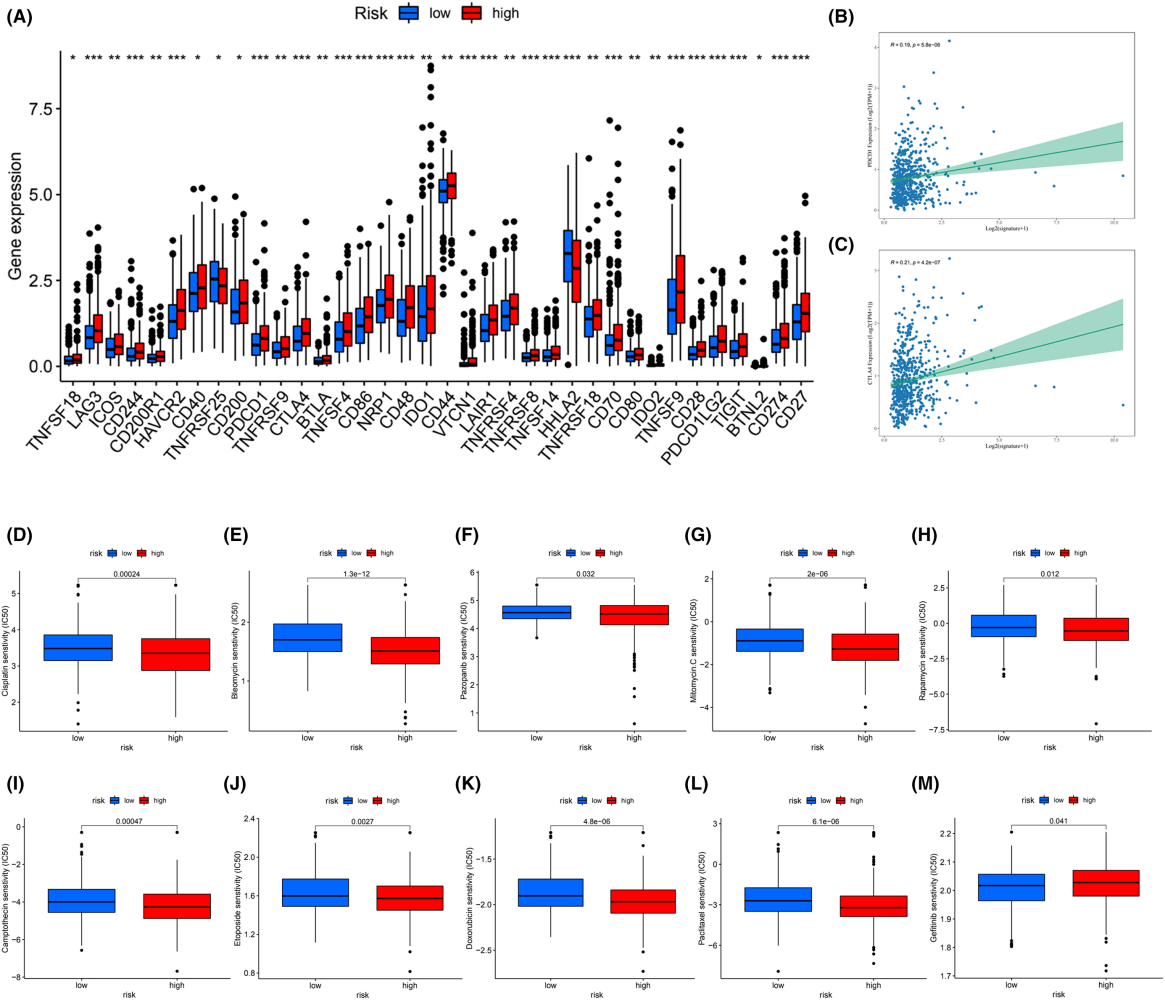
Treatment of LRGS. (A) Differences in immune checkpoint expression between high‐risk and low‐risk groups. (B) Association between LRGS and PD1 expression. (C) Association between LRGS and CTLA4 expression. *p < 0.05; **p < 0.01; ***p < 0.001; Ns, no sense. Sensitivity analysis of different drugs in patients with high and low risk. (D) Cisplatin, (E) bleomycin, (F) pazopanib, (G) mitomycin, (H) rapamycin, (I) camptothecin, (J) etoposide, (K) doxorubicin, (L) paclitaxel, and (M) gefitinib.

Immune checkpoint inhibitor (ICI) therapy is the most promising treatment in oncology. We analyzed the correlation between LRGS and 47 ICI‐related biomarkers. The results indicated that 36 ICI‐related biomarkers were differentially denoted in the two groups; of them, 35 presented upregulation in the high‐risk group, and only HHLA2 was upregulated in the low‐risk group (Figure 9A). PD‐1 and CTLA‐4 inhibitors are the two most extensively used ICIs. We found a positive connection between the expression levels of the two genes and the risk score (Figure 9B,C). According to the obtained data, patients in the high‐risk group can exhibit a higher immune response to immunotherapy, resulting in improved patient outcomes.

Chemotherapy is usually used in the treatment of metastatic CRC. With the purpose of comparing the difference in chemotherapy drug sensitivity between the two groups, the pRRophetic algorithm was adopted for calculating the IC50 of the two groups. This study attempted to compare the estimated IC50 levels of 138 chemotherapeutic agents or inhibitors in the two groups. The results of 10 representative drugs are presented in Figure 9D–M. The results revealed that cisplatin, bleomycin, pazopanib, mitomycin, rapamycin, camptothecin, etoposide, doxorubicin, and paclitaxel may be candidates for treating patients in the low‐risk group. However, gefitinib may not be appropriate for low‐risk patients. Therefore, risk scores can be used in identifying patients with different levels of risk, contributing to delivering appropriate treatment options.

## 4 DISCUSSION

Advanced CRC is often difficult to treat.^40^ Traditional surgical resection and chemotherapy have limited therapeutic effects in patients with advanced CRC. Because of the scientific research and clinical development in recent years, targeted therapy and immunotherapy have emerged as the new treatment options for patients with advanced CRC.^41^, ^42^ However, both therapies have certain limitations. Targeted therapies, including cetuximab‐mediated inhibition of epidermal growth factor, should only be used in patients with specific subtypes of CRC. ICIs are also only suitable for repairing CRC with mismatch defects and high microsatellite instability, and unsatisfactory clinical efficacy is observed in most patients. Recently, metabolic reprogramming has been recognized as a hallmark of cancer, and cancer cells often reprogram their metabolism because of their high energy and nutrient requirements.^43^ Therefore, various metabolic abnormalities, such as glycolysis, glutamine decomposition, lipid synthesis, and mitochondrial oxidation, are often detected in tumors. During the metabolic reprogramming of cancer cells, the TME undergoes metabolic rewiring to support cancer cell proliferation, metastasis, and escape from immune surveillance.^44^ Therefore, several scholars have demonstrated that the use of metabolic targeting in TME is a potential treatment strategy for cancer.^45^ Lactic acid is a vital component of tumor metabolism and has a vital role in reprogrammed TME. Therefore, the current study attempted to build a prognostic LRGS model with the application of LRGs, aiming to determine the prognosis of patients suffering from CRC, compare the differences between TMB and immune infiltration, and select appropriate immunotherapy or chemotherapy drugs.

In this study, totally 153 differentially expressed LRGs were detected by comparing the expression of tumor and normal tissues in patients with CRC. This work extracted the data of patients from the TCGA database. The GO and KEGG enrichment analyses of these genes revealed their involvement in respiration, energy synthesis, metabolism, and other related processes. Thus, tumor cells can produce lactic acid through a variety of metabolic pathways, such as glycolysis, and lactic acid is also an energy source for tumor cells.^46^ With the purpose of predicting the prognosis of patients undergoing CRC, this study employed LASSO and Cox regression to construct the LRGS model. Four genes were used to construct the LRGS; of them, *CPT2*, *MIPEP*, and *NFS1* are protective genes, and *ISCU* is a risk gene. The median risk score was applied to categorize patients into the high‐risk and low‐risk groups. Apart from that, the predictive power of LRGS was confirmed using survival analysis and ROC in three different cohorts. The subgroup survival analysis also confirmed the effectiveness. Furthermore, we constructed a nomogram by using LRGS and clinically important case parameters (age, sex, and tumor stage). The constructed nomogram accurately predicted the survival at 1, 3, and 5 years for patients with CRC, which facilitates the use of LRGS. The results of GSEA indicated that ECM, KRAS signaling, and angiogenesis were enriched in the high‐risk group. Therefore, lactic acid may promote tumor angiogenesis by signaling through the EMT pathway or by activating KRAS, leading to tumor progression.

*CPT2*, *MIPEP*, *NFS1*, and *ISCU* were the four genes used to build the model. Each of the four genes has previously been linked to cancer or metabolism. *CPT2*, which encodes proteins that oxidize long‐chain fatty acids in mitochondria, is essential for fatty acid metabolism,^47^ and downregulation of *CPT2* expression has been observed in several tumors compared to normal tissues.^48^, ^49^ Downregulation of *CPT2* stimulates tumor proliferation in colorectal cancer via the p53 pathway.^50^ Furthermore, the downregulation of *CPT2* in colorectal cancer induces stemness and drug resistance by activating glycolytic metabolism induced by the ROS/Wnt/β‐linked protein pathway.^51^ *MIPEP*, a mitochondrial intermediate peptidase, is primarily involved in the maturation of proteins involved in oxidative phosphorylation. *MIPEP* expression is higher in more malignant breast cancers.^52^ *NFS1* is a rate‐limiting enzyme in iron–sulfur cluster biosynthesis that is overexpressed in colorectal cancer compared to normal tissue and has been linked to a poor prognosis.^53^ *ISCU*, an iron–sulfur cluster assembly enzyme, is required in mitochondria to synthesize iron–sulfur (Fe‐S) cluster scaffold proteins. *ISCU* is involved in tumorigenesis and progression in various cancers by being regulated by miR‐210.^54^, ^55^ Based on our GSEA results and current findings, we examine multiple pathways for these four genes to influence colorectal cancer progression, but more research is needed to investigate them.

TME is consisted of tumor cells, stromal cells, immune cells, and ECM. TME can influence the progression of CRC in a number of ways, including blood vessel growth, ECM remodeling, and the activation and recruitment of immune cells.^56^ Obviously, the various kinds of immune cells present in TME play distinct roles in the antitumor and immune escape processes. Therefore, tumor‐associated immune cells may have tumor antagonistic as well as tumor‐promoting roles.^57^ We discovered that patients at high and low risk had distinct stromal and immunological components and tumor purity. We used several immunological algorithms to demonstrate an obvious difference in immune infiltration between the two groups of patients with CRC. A previous study revealed that diffuse carcinoma has six distinct immunological subtypes.^58^ Patients suffering from the C3 subtype had the best prognosis, whereas patients with the C6 subtype had the worst. Moreover, the high‐risk group had a higher proportion of patients with the C6 subtype and a poorer prognosis, whereas the low‐risk group had a higher proportion of patients with the C3 subtype as well as a better outcome. This finding supports our previous results.

Immunotherapy, a promising treatment method, is gradually becoming the main treatment for a variety of tumors. CRC can be categorized into the following two types: tumors with mismatch repair deficiency and high microsatellite instability (dMMR‐MSI‐H) and tumors with proficient mismatch repair or low microsatellite instability (pMMR‐MSI‐L). ICI therapy had a superior therapeutic effect on patients suffering from dMMR‐MSI‐H. It is difficult to determine the optimal immunotherapy regimen for an individual. PD‐1 and CTLA‐4 inhibitors are the most extensively used ICIs. A previous study suggested that TMB can be utilized as a biomarker to predict the efficacy of ICI.^59^ In this study, LRGS was positively associated with TMB; therefore, the high‐risk group may exhibit a superior immunotherapy response. In addition, this study also identified higher expression levels of *PD‐1* and *CTLA‐4* in the high‐risk group. Thus, PD‐1 and CTLA‐4 inhibiting drugs elicit a superior response in patients with high LRGS scores.

Chemotherapy remains the primary treatment for patients with CRC, and the sensitivity to chemotherapy medicines influences the treatment efficacy. According to the statistical results of several studies, a number of chemotherapeutic drugs are effective in the treatment of CRC. However, many patients are less sensitive to drugs, thereby limiting the efficacy of chemotherapy. The common chemotherapy medication, cisplatin, can reduce NADPH levels in CRC cells.^60^ The injection of bleomycin into mice colon cancer cells using electrical pulses killed cancer cells and stimulated the immune system.^61^ Pazopanib induced PUMA expression in colon cancer cells, thereby promoting apoptosis.^62^ The combination of surgical resection and mitomycin is an effective treatment for metastatic CRC.^63^ Rapamycin can reduce TLR4 expression, thereby demonstrating an antitumor effect.^64^ Camptothecin is effective against several cancers, including CRC.^65^ Etoposide can be used to treat CRC with metastases.^66^ Adriamycin can limit the proliferation of CRC cells by blocking DNA replication.^67^ Paclitaxel is also an effective treatment for CRC with metastases.^68^ Gefitinib reduced the viability of CRC cells in vitro.^69^ According to our study, the low‐risk group had more sensitivity to medications other than gefitinib and may respond better to chemotherapy. Therefore, the sensitivity of patients to chemotherapeutic agents can be predicted using the LRGS, and clinicians can use the predictions to provide individualized therapy recommendations.

Our study has some limitations. First, the genes used in the construction of LRGS are not key genes in lactate metabolism, and more experiments are required to discover their mechanisms. In addition, our model is not efficient in determining the 5‐year survival rate of patients, which may be because of the incomplete LRGs included in this study. Moreover, the results of this study should be validated using other independent databases.

To conclude, this study evaluated the prognostic significance of LGRS in CRC, its impact on TME, response to ICI therapy, and sensitivity to chemotherapeutic agents. Risk stratification based on lactic acid‐related prognostic characteristics showed negative relationship to the clinical prognosis of patients. Apart from that, the model suggests that patients with higher risk scores may benefit more from ICI therapy, while patients in the lower risk group may have better sensitivity to pharmacotherapeutic agents. Overall, the obtained results may help to illustrate the function of lactate in TME of CRC. In summary, the reconstructed prognostic profile may be applied clinically with the aim of improving survival and provide a target for future cures in patients with CRC.

## AUTHOR CONTRIBUTIONS

**Zhi Tong:** Resources (lead); software (equal); validation (lead); visualization (lead); writing – original draft (equal). **Xinyu Wang:** Resources (supporting); writing – original draft (supporting). **Sanbao Shi:** Software (supporting); validation (supporting). **Tiewei Hou:** Resources (supporting); supervision (supporting). **Guangrong Gao:** Supervision (supporting); writing – original draft (supporting). **Da Li:** Software (supporting); supervision (supporting). **Yongqi Shan:** Resources (supporting); software (supporting); validation (supporting). **Cheng Zhang:** Supervision (lead); writing – review and editing (lead).

## CONFLICT OF INTEREST STATEMENT

The authors declare no conflict of interest.

## ETHICS APPROVAL AND CONSENT TO PARTICIPATE

The ethics committee approved the study of the General Hospital of Northern Theater Command (Shenyang, Liaoning, P.R. China) and the IRB approval number is Y (2022) 063.

## INFORMED CONSENT STATEMENT

Not applicable.

## Supporting information


Figure S1.

Figure S2.

Table S1.

## Data Availability

The original contributions presented in the study are included in the article/Supplementary Material. Further inquiries can be directed to the corresponding authors.
