# Supplementary figures and images for: Development of lactate‐related gene signature and prediction of overall survival and chemosensitivity in patients with colorectal cancer
Source: Cancer Med. 2023 Feb 12;12(8):10105–22. doi: 10.1002/cam4.5682 (PMC10166923; doi:10.1002/cam4.5682)

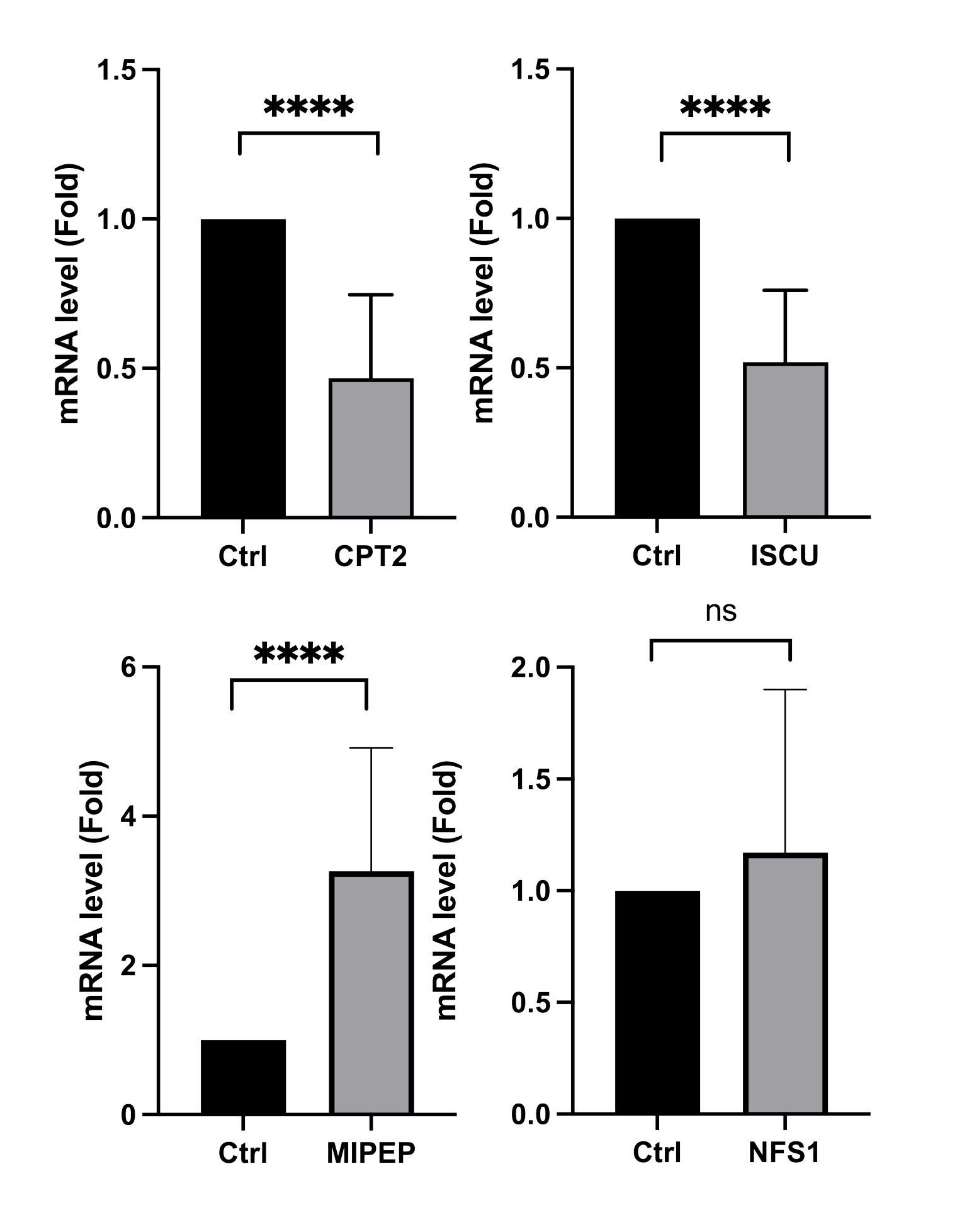

Supplement: Supplementary file 1 — Figure S1. [file CAM4-12-10105-s002.tif]

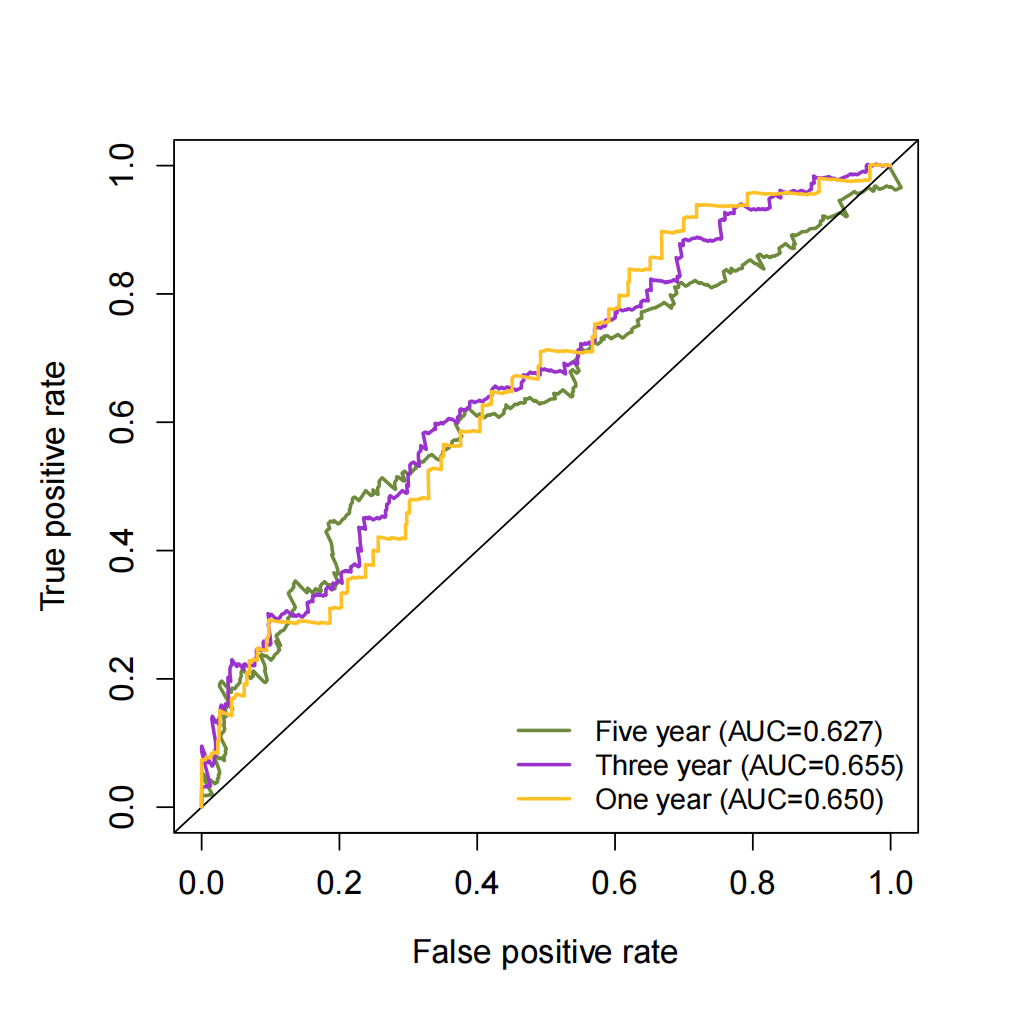

Supplement: Supplementary file 2 — Figure S2. [file CAM4-12-10105-s001.tif]
