# Supplementary material for: Development of lactate‐related gene signature and prediction of overall survival and chemosensitivity in patients with colorectal cancer
Source: Cancer Med. 2023 Feb 12;12(8):10105–22. doi: 10.1002/cam4.5682 (PMC10166923; doi:10.1002/cam4.5682)
Supplement: Supplementary file 3 — Table S1. [file CAM4-12-10105-s003.docx]

Supplementary Materials

**SUPPLEMENTARY TABLE 1.** Sequences of primers included in this study.

| **Genes** | **Forward primer (5’-3’)** | **Reverse primer (5’-3’)** |
| --- | --- | --- |
| CPT2 | TCCGTCTATACAAAGAGGTGC | CAAACAAGTGTCGGTCAAA |
| ISCU | ATGGGTGAAAGGAAAGACG | AACAGCACAGATTTGGAGC |
| MIPEP | GCCGGGGTCTTTTTGGAGT | GCACGGTCCACAAGCAATTC |
| NFS1 | GTGGCCCGATTCTACAGGTC | TTCTGCACTGGGAGGTAGGT |
| GAPDH | TGGACCTGACCTGCCGTCTA | AGGAGTGGGTGTCGCTGTTG |

**
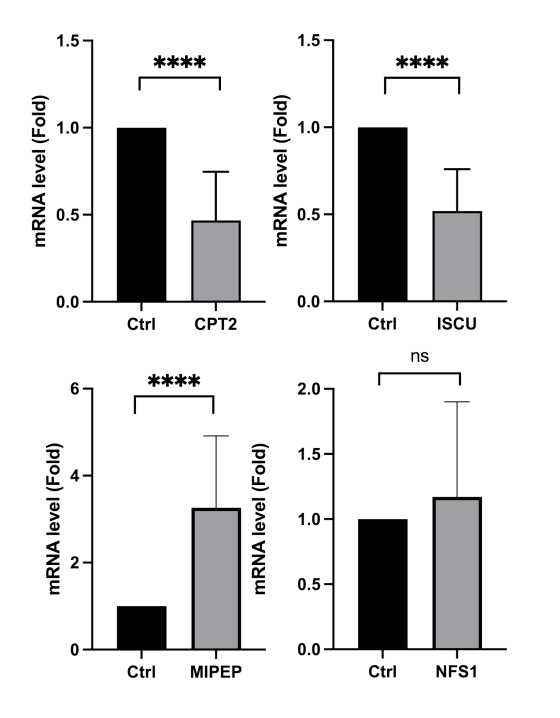
**

**SUPPLEMENTARY FIGURE 1** The expression levels of four target genes by qRT-PCR.


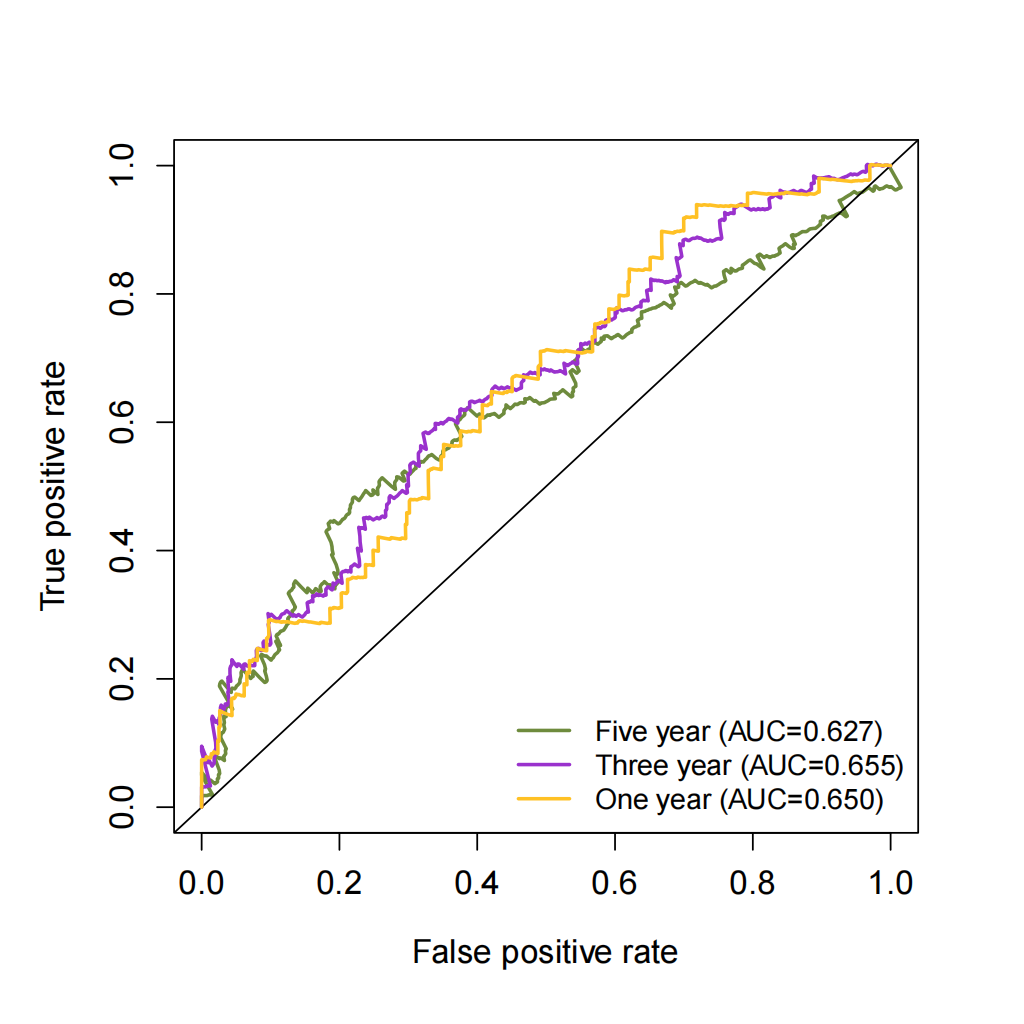


**SUPPLEMENTARY FIGURE 2** ROC curve analysis of LRGS at 1, 3, and 5 years in the training and validation set.
